# Distinct Associations of Age and CYP2D6 Genotype With Parent Venlafaxine Exposure and O‐Demethylation in Japanese Psychiatric Patients

**DOI:** 10.1002/npr2.70175

**Published:** 2026-10-29

**Authors:** Yugo Ishihara, Taro Sasaki, Tomohiro Asahi, Yujin Ono, Tomomi Sugisaki, Rina Hiramatsu, Yasushi Kawamata, Norio Sugawara, Norio Yasui‐Furukori

**Affiliations:** ^1^ Department of Psychiatry Dokkyo Medical University School of Medicine Tochigi Japan

**Keywords:** aging, CYP2D6, O‐desmethylvenlafaxine, pharmacogenetics, therapeutic drug monitoring, venlafaxine

## Abstract

**Background:**

Venlafaxine (VEN) is converted to O‐desmethylvenlafaxine (ODV) mainly through CYP2D6‐mediated O‐demethylation. However, age‐related clinical factors may also influence VEN exposure in routine practice. We examined whether age and CYP2D6 genotype show different associations with parent‐drug exposure and metabolic conversion.

**Methods:**

This retrospective therapeutic drug monitoring analysis included 78 Japanese psychiatric patients with available plasma VEN and ODV concentrations and CYP2D6 genotype data. Two prespecified outcomes were analyzed without body‐weight correction: VEN concentration divided by daily dose (VEN/dose) and the ODV/VEN ratio. CYP2D6*10 and *5 were coded as reduced‐ and no‐function alleles, respectively. Log‐transformed outcomes were analyzed using regression models including age and CYP2D6 reduced/no‐function allele count.

**Results:**

Nineteen patients (24.4%) were aged 65 years or older. Age was correlated with VEN/dose (Spearman rho = 0.295, *p* = 0.0086), and older patients had higher VEN/dose than younger patients (median, 0.522 vs. 0.192 [ng/mL]/[mg/day], *p* = 0.0127). In regression analysis, the incremental R^2^ for age was numerically larger than that for CYP2D6 allele count for VEN/dose (incremental R^2^, 0.058 vs. 0.026). Conversely, the ODV/VEN ratio was strongly associated with CYP2D6 allele count (standardized beta = −0.391, *p* < 0.001; incremental R^2^ = 0.145) but not with age. Age‐by‐genotype interactions were not significant for either outcome.

**Conclusions:**

Age appeared to be more closely related to dose‐corrected parent VEN exposure, whereas CYP2D6 genotype predominantly determined the ODV/VEN metabolic ratio. The relative contribution of age should be interpreted descriptively because no formal comparison of effect sizes was performed. Separating exposure and metabolic‐ratio outcomes may clarify how geriatric and pharmacogenetic factors contribute to VEN disposition.

## Abstract 2

This TDM study suggests that, in Japanese psychiatric patients receiving venlafaxine, age is associated with dose‐corrected parent‐drug exposure, whereas CYP2D6 genotype predominantly determines the ODV/VEN metabolic ratio.

## Graphical abstract

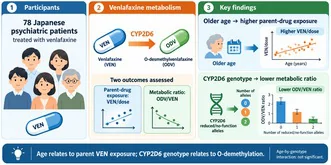

## 1 Introduction

Venlafaxine (VEN) is a serotonin‐norepinephrine reuptake inhibitor used for depressive and anxiety disorders. Its major active metabolite, O‐desmethylvenlafaxine (ODV), is formed primarily by CYP2D6‐mediated O‐demethylation. Accordingly, CYP2D6 genotype can influence the balance between the parent drug and its active metabolite, particularly in carriers of reduced‐ or no‐function alleles [1, 2].

Therapeutic drug monitoring (TDM) is clinically relevant for VEN because plasma concentrations vary widely between patients [3, 4]. Japanese pharmacogenetic studies have shown that CYP2D6*10 and *5 influence VEN metabolism and ODV/VEN ratios [5, 6, 7]. In parallel, large TDM datasets suggest that dose‐corrected concentrations of several antidepressants, including VEN, increase with age, indicating that non‐genetic clinical factors may be important in older patients [8, 9].

A clinically useful question is whether age and CYP2D6 genotype affect the same pharmacokinetic outcome or different aspects of VEN disposition. We therefore examined two outcomes: dose‐corrected parent VEN concentration as an index of parent‐drug exposure, and the ODV/VEN ratio as an index of CYP2D6‐mediated O‐demethylation. We hypothesized that age would be more strongly associated with VEN/dose, whereas CYP2D6 genotype would be more strongly associated with the ODV/VEN ratio.

## 2 Methods

This retrospective TDM analysis used deidentified records from Japanese psychiatric patients who were prescribed VEN. Records were eligible when age, daily VEN dose, plasma VEN concentration, plasma ODV concentration, and CYP2D6 genotype were available. When multiple samples were available for the same patient, the latest sample was selected to avoid within‐patient duplication. The study was approved by Dokkyo Medical University Hospital Ethics Committee for Clinical Trials (approval number: R‐109‐1 J), and written informed consent was waived with an opt‐out opportunity according to the approved protocol.

The TDM and genotyping procedures followed previously reported methods [6]. In brief, blood sampling was performed after at least 1 week of stable VEN dosing to approximate steady‐state exposure. Venous blood was collected 9–13 h after the previous evening dose into heparinized tubes, and plasma was separated by centrifugation and stored at −80 degrees C until measurement. Plasma VEN and ODV concentrations were quantified using high‐performance liquid chromatography with ultraviolet detection and mirtazapine as the internal standard; the assay was based on a published method with local modifications [6, 10]. The lower limit of quantification was 2.5 ng/mL for both analytes.

Genomic DNA was extracted from the cellular fraction of blood samples. CYP2D6*1, CYP2D6*2, and CYP2D6*10 were identified using mutation‐specific polymerase chain reaction, and the CYP2D6*5 deletion allele was detected using long‐polymerase chain reaction [6, 11, 12]. For the present analysis, CYP2D6*10 was treated as a reduced‐function allele and CYP2D6*5 as a no‐function allele, whereas CYP2D6*1 and *2 were treated as normal‐function alleles. The number of reduced‐ or no‐function alleles was coded as 0, 1, or 2.

Two pharmacokinetic outcomes were prespecified. Parent‐drug exposure was calculated as plasma VEN concentration (ng/mL) divided by daily VEN dose (mg/day). The metabolic ratio was calculated as plasma ODV concentration divided by plasma VEN concentration. Body‐weight correction was not used in the primary analysis because the aim was to evaluate a clinically intuitive dose‐corrected index corresponding to routine prescribing. Concentrations recorded at the lower limit of quantification were retained in the primary analysis; a sensitivity analysis excluded records in which both VEN and ODV were at the lower limit of quantification.

Continuous variables are reported as medians with interquartile ranges (IQRs). Spearman correlation, Mann–Whitney *U* tests, and Kruskal–Wallis tests were used for univariable analyses. Because both outcomes were right‐skewed, multivariable linear regression was performed using log‐transformed outcomes. Age and CYP2D6 reduced/no‐function allele count were entered simultaneously. Standardized beta coefficients and incremental R^2^ values were used to describe the relative explanatory contribution of age and CYP2D6 reduced/no‐function allele count. No formal statistical test was performed to compare these effect‐size indices directly; therefore, differences in incremental R^2^ were interpreted descriptively and cautiously. Age‐by‐CYP2D6 interaction terms were examined for both outcomes. All tests were two‐sided, and *p* < 0.05 was considered statistically significant.

## 3 Results

The analysis included 78 patients. The median age was 48.0 years (IQR, 34.3–63.5; range, 20–84), and 19 patients (24.4%) were aged 65 years or older. The median daily VEN dose was 75.0 mg/day (IQR, 37.5–150.0). CYP2D6 reduced/no‐function allele counts were 0, 1, and 2 in 36, 28, and 14 patients, respectively (Table 1).

**TABLE 1:** Patient characteristics and pharmacokinetic outcomes.

Characteristic	Overall (*n* = 78)
Age, years	48.0 (34.3–63.5) (20–84)
Age > = 65 years	19 (24.4%)
Daily VEN dose, mg/day	75.0 (37.5–150.0) (37.5–225.0)
VEN concentration, ng/mL	17.5 [7.4–54.0] (2.5–262.7)
ODV concentration, ng/mL	55.7 (17.1–99.8) (2.5–491.6)
VEN/dose, (ng/mL)/(mg/day)	0.216 (0.114–0.506) (0.017–2.687)
ODV/VEN ratio	2.74 (1.08–5.12) (0.197–19.53)
CYP2D6 reduced/no‐function allele count: 0	36 (46.2%)
CYP2D6 reduced/no‐function allele count: 1	28 (35.9%)
CYP2D6 reduced/no‐function allele count: 2	14 (17.9%)

*Note:* Values are median (interquartile range) (range) or *n* (%).

Abbreviations: ODV, O‐desmethylvenlafaxine; VEN, venlafaxine.

Age was positively correlated with VEN/dose (Spearman rho = 0.295, *p* = 0.0086). Patients aged 65 years or older had higher VEN/dose than those aged < 65 years (median, 0.522 vs. 0.192 [ng/mL]/[mg/day], *p* = 0.0127). In the regression model including age and CYP2D6 allele count, age remained significantly associated with higher VEN/dose (standardized beta = 0.248, *p* = 0.0299; 10‐year multiplicative effect, 1.16), whereas CYP2D6 allele count was not statistically significant (standardized beta = 0.167, *p* = 0.1399; per‐allele multiplicative effect, 1.27). The incremental R^2^ was numerically larger for age than for CYP2D6 allele count (0.058 vs. 0.026; Table 2).

**TABLE 2:** Relative impact of age and CYP2D6 genotype on the two pharmacokinetic outcomes.

Outcome	Predictor	Std beta	Multiplicative effect	*p*	Incremental R^2^
VEN/dose	Age	0.248	1.16 per 10 years	0.0299	0.058
VEN/dose	CYP2D6 allele count	0.167	1.27 per allele	0.1399	0.026
ODV/VEN ratio	Age	−0.043	0.98 per 10 years	0.6953	0.002
ODV/VEN ratio	CYP2D6 allele count	−0.391	0.59 per allele	0.00056	0.145

*Note:* Regression models used log‐transformed outcomes and included age and CYP2D6 reduced/no‐function allele count simultaneously. The age‐by‐CYP2D6 interaction was not significant for VEN/dose (*p* = 0.867) or ODV/VEN ratio (*p* = 0.526).

The ODV/VEN ratio differed across CYP2D6 allele‐count groups (Kruskal–Wallis *p* = 0.00054). Median ODV/VEN ratios were 4.15, 1.54, and 1.46 in patients with 0, 1, and 2 reduced/no‐function alleles, respectively. In the regression model, CYP2D6 allele count was strongly associated with a lower ODV/VEN ratio (standardized beta = −0.391, *p* = 0.00056; per‐allele multiplicative effect, 0.59; incremental R^2^ = 0.145), whereas age was not (standardized beta = −0.043, *p* = 0.6953; incremental R^2^ = 0.002).

Age‐by‐CYP2D6 interaction terms were not significant for VEN/dose (*p* = 0.867) or ODV/VEN ratio (*p* = 0.526), indicating no statistical evidence that CYP2D6 effects differed by age group in this dataset. In a sensitivity analysis excluding three records in which both VEN and ODV concentrations were at the lower limit of quantification, the direction of findings was unchanged: age remained correlated with VEN/dose (Spearman rho = 0.267, *p* = 0.021), and the ODV/VEN ratio remained significantly different across CYP2D6 allele‐count groups (Kruskal–Wallis *p* = 0.00064).

## 4 Discussion

This retrospective TDM analysis indicates that age and CYP2D6 genotype are linked to different aspects of VEN pharmacokinetics. Age showed a significant association with dose‐corrected parent VEN concentration and a numerically larger incremental R^2^ than CYP2D6 allele count, whereas CYP2D6 genotype was the main determinant of the ODV/VEN ratio. Because no formal statistical comparison between these effect‐size indices was performed, this difference should be interpreted as descriptive and hypothesis‐generating. The association between age and VEN/dose should also be interpreted cautiously. Chronological age itself is unlikely to be the sole causal determinant of increased parent‐drug exposure. Rather, age may act as a clinical proxy for multiple factors, including renal function, hepatic blood flow, body composition, comorbidities, adherence, and concomitant medications. Therefore, the present findings suggest the importance of age‐related clinical assessment, rather than supporting dose adjustment based on chronological age alone. This distinction is clinically important because parent‐drug exposure and metabolic conversion are related but not interchangeable pharmacokinetic constructs.

The stronger association between age and VEN/dose is consistent with the clinical observation that older patients may have higher dose‐corrected antidepressant concentrations [8, 9, 13]. This finding should not be interpreted as a direct causal effect of chronological age alone. Age may capture multiple clinical factors that were not fully modeled here, including renal function, hepatic blood flow, body composition, comorbidity, adherence, and concomitant medications. Thus, VEN/dose appears to reflect an integrated exposure phenotype in routine practice rather than a purely genetic phenotype.

By contrast, the ODV/VEN ratio was clearly sensitive to CYP2D6 allele count. This supports the use of the ODV/VEN ratio as a functional marker of CYP2D6‐mediated O‐demethylation in VEN‐treated patients [1, 14]. The result also aligns with previous pharmacogenetic studies showing reduced conversion from VEN to ODV in carriers of CYP2D6 reduced‐ or no‐function alleles [5, 6, 7]. Importantly, the nonsignificant age‐by‐genotype interaction does not support the conclusion that CYP2D6 effects are attenuated in older patients. A more appropriate interpretation is that CYP2D6 effects were most visible when the outcome directly represented O‐demethylation, whereas age‐related factors were most visible when the outcome represented parent‐drug exposure.

These findings have practical implications for individualized VEN therapy. Pharmacogenetic information may help interpret reduced O‐demethylation, whereas TDM can capture the combined consequences of age, organ function, comedication, adherence, and genotype on actual drug exposure. In older patients, relying on genotype alone may overlook non‐genetic factors that increase parent‐drug concentrations. Conversely, TDM results without genotype information may not fully explain an unusually low ODV/VEN ratio. Combining TDM with pharmacogenetic and geriatric assessment may therefore provide a more complete basis for dose optimization.

Several limitations should be acknowledged. The sample size was modest, especially among patients aged 65 years or older. The retrospective design limited control over sampling conditions, adherence, and concomitant medications. The primary models were intentionally parsimonious and did not adjust for sex, body weight, renal function, hepatic function, or CYP2D6 inhibitor use. The genotyping panel was limited to alleles available in the dataset and did not capture all reduced‐function, no‐function, or copy‐number variants. Clinical outcomes, adverse events, and total active moiety exposure were not evaluated as primary endpoints. Therefore, the findings should be considered hypothesis‐generating and require confirmation in larger prospective cohorts.

In conclusion, separating VEN pharmacokinetics into parent‐drug exposure and metabolic‐ratio outcomes clarified different patterns of association with age and CYP2D6 genotype. Age showed a significant association and a numerically larger incremental R^2^ for dose‐corrected parent VEN exposure, whereas CYP2D6 genotype showed a stronger and more specific association with the ODV/VEN ratio. These findings support integrating TDM, pharmacogenetic information, and geriatric clinical assessment when optimizing VEN treatment in psychiatric practice.

## Author Contributions

Yugo Ishihara: conceptualization, formal analysis, visualization, and writing – original draft. Taro Sasaki: data curation, investigation, methodology, and writing – review and editing. Tomohiro Asahi, Yujin Ono, Tomomi Sugisaki, and Rina Hiramatsu: data curation, investigation, and writing – review and editing. Yasushi Kawamata: clinical interpretation, supervision, and writing – review and editing. Norio Sugawara: methodology, statistical supervision, validation, and writing – review and editing. Norio Yasui‐Furukori: conceptualization, methodology, project administration, supervision, funding acquisition, and writing – review and editing. All authors approved the final version of the manuscript and agree to be accountable for all aspects of the work.

## Funding

This work was supported by departmental research funds from the Department of Psychiatry, Dokkyo Medical University. NY‐F has received public research funding from the Japan Agency for Medical Research and Development (AMED), Grants‐in‐Aid for Scientific Research (KAKENHI), and Health and Labour Sciences Research Grants from the Ministry of Health, Labour and Welfare of Japan. The funding sources had no role in the study design; data collection, analysis, or interpretation; preparation of the manuscript; or the decision to submit the article for publication.

## Ethics Statement

The study was approved by Dokkyo Medical University Hospital Ethics Committee for Clinical Trials (approval number: R‐109‐1 J), and written informed consent was waived with an opt‐out opportunity according to the approved protocol.

## Conflicts of Interest

N.Y.‐F. reports having received honoraria for lectures from Daiichi Sankyo, EA Pharma, Eisai, Eli Lilly and Company, Hisamitsu Pharmaceutical, Janssen Pharmaceuticals, Kowa, Lundbeck, Meiji Seika Pharma, Mitsubishi Tanabe Pharma, Mochida Pharmaceutical, MSD, Ono Pharmaceutical, Otsuka Pharmaceutical, Shionogi, Sumitomo Pharma, Takeda Pharmaceutical, Tsumura, Viatris, and Yoshitomi Yakuhin; has served in advisory or consultancy roles for EA Pharma, Bristol Myers Squibb, Boehringer Ingelheim, Janssen Pharmaceuticals, Mitsubishi Tanabe Pharma, Mochida Pharmaceutical, Sumitomo Pharma, and Otsuka Pharmaceutical; and has participated as a sub‐investigator in industry sponsored clinical trials conducted by EA Pharma and Bristol Myers Squibb. The remaining authors declare that they have no known competing financial interests or personal relationships that could have appeared to influence the work reported in this paper.

## Supporting information


**Data S1:** Analysis script for data processing, statistical analyses, sensitivity analyses, and Figure 1 generation.

**Table S1:** Variable definitions for the controlled‐access analytical dataset.

## Data Availability

Patient‐level analytical data are not publicly deposited because unrestricted public release of clinical therapeutic drug monitoring data combined with CYP2D6 genotype information was outside the scope of the approved ethics protocol and opt‐out arrangements, and may carry a residual risk of re‐identification in this small single‐institution dataset. A minimum deidentified analytical dataset underlying Tables 1, 2 and Figure 1, including age, daily venlafaxine dose, venlafaxine and O‐desmethylvenlafaxine concentrations, CYP2D6 genotype, reduced/no‐function allele‐count coding, derived VEN/dose and ODV/VEN variables, and a flag for records excluded from the sensitivity analysis, can be made available to qualified researchers through controlled access. Requests should be submitted to the corresponding author with a brief research proposal and will require approval by Dokkyo Medical University Hospital or confirmation of appropriate ethics approval, as well as a data use agreement prohibiting re‐identification, redistribution, and use beyond the approved purpose. The analysis script used for data processing, statistical analyses, sensitivity analyses, and Figure 1 generation is provided as a separate Supporting Information file, Data S1 (Data_S1_analysis_script.docx). Variable definitions for the controlled‐access analytical dataset are provided as a separate Supporting Information file, Table S1 (Table_S1_Variable_Definitions.docx). Neither file contains patient‐level data or identifying information. Outcome‐specific associations of age and CYP2D6 genotype. (A) Age was positively associated with dose‐corrected parent venlafaxine concentration (VEN/dose). (B) The ODV/VEN ratio was lower in carriers of CYP2D6 reduced‐ or no‐function alleles. Outcomes are shown on logarithmic scales.
