# Supplementary material for: Distinct Associations of Age and CYP2D6 Genotype With Parent Venlafaxine Exposure and O‐Demethylation in Japanese Psychiatric Patients
Source: Neuropsychopharmacol Rep. 2026 Oct 29;46(4):e70175. doi: 10.1002/npr2.70175 (PMC13719267; doi:10.1002/npr2.70175)
Supplement: Supplementary file 1 [file NPR2-46-e70175-s001.docx]

**Data S1**

*Analysis script for data processing, statistical analyses, sensitivity analyses, and Figure 1 generation*

**Note: This file contains analysis code only. It does not contain patient-level data or identifying information.**

```
#!/usr/bin/env python3
"""
Supplementary Code S1

Manuscript:
Distinct associations of age and CYP2D6 genotype with parent venlafaxine exposure
and O-demethylation in Japanese psychiatric patients.

Purpose:
This script reproduces the data processing, descriptive summaries, regression
analyses, sensitivity analyses, and Figure 1 generation described in the manuscript.
It is intended to be run only with the controlled-access deidentified analytical
dataset described in the Data Availability Statement.

Required input file:
  VEN_minimum_deidentified_analytical_dataset_controlled_access.csv

The patient-level analytical dataset is not included in this Supporting Information
file because public release of clinical therapeutic drug monitoring data combined
with CYP2D6 genotype information is outside the approved ethics/opt-out framework.
This script contains no patient-level data and no identifying information.

How to run:
  python Supplementary_Code_S1_analysis_script.py \
    --input VEN_minimum_deidentified_analytical_dataset_controlled_access.csv \
    --outdir analysis_outputs

Expected outputs:
  table1_summary.csv
  table2_regression_results.csv
  interaction_results.csv
  sensitivity_summary.txt
  figure1_outcome_specific_associations.png

Software versions used for validation:
  Python 3.13.5
  pandas 2.2.3
  numpy 2.3.5
  scipy 1.17.0
  statsmodels 0.14.6
  matplotlib 3.10.8

Required input variables are defined in Supplementary Table S1.
"""

import argparse
import sys
import platform
from pathlib import Path

import numpy as np
import pandas as pd
import scipy
from scipy import stats
import statsmodels
import statsmodels.api as sm
import matplotlib
matplotlib.use("Agg")
import matplotlib.pyplot as plt


def multiplicative_effect(model, variable, scale=1.0):
    """Return exp(beta * scale) from a log-linear model."""
    return float(np.exp(model.params[variable] * scale))


def incremental_r2(df, outcome_col, predictors_full, predictor_to_remove):
    """Calculate incremental R2 from removing one predictor from a full model."""
    y = np.log(df[outcome_col].astype(float))
    X_full = sm.add_constant(df[predictors_full].astype(float), has_constant="add")
    full = sm.OLS(y, X_full).fit()
    reduced_predictors = [p for p in predictors_full if p != predictor_to_remove]
    X_reduced = sm.add_constant(df[reduced_predictors].astype(float), has_constant="add")
    reduced = sm.OLS(y, X_reduced).fit()
    return float(full.rsquared - reduced.rsquared), full


def standardized_beta(df, outcome_col, predictors):
    """Fit a model using standardized predictors and log-transformed outcome."""
    tmp = df[[outcome_col] + predictors].astype(float).copy()
    y = np.log(tmp[outcome_col])
    y_z = (y - y.mean()) / y.std(ddof=0)
    X = tmp[predictors].copy()
    X_z = (X - X.mean()) / X.std(ddof=0)
    X_z = sm.add_constant(X_z, has_constant="add")
    return sm.OLS(y_z, X_z).fit()


def median_iqr_range(series, decimals=3):
    """Return median, IQR, and range as rounded values."""
    s = series.dropna().astype(float)
    return {
        "median": round(float(s.median()), decimals),
        "q1": round(float(s.quantile(0.25)), decimals),
        "q3": round(float(s.quantile(0.75)), decimals),
        "min": round(float(s.min()), decimals),
        "max": round(float(s.max()), decimals),
    }


def create_table1(df, outdir):
    """Create Table 1 descriptive summary."""
    summary_rows = []
    continuous_variables = [
        ("Age, years", "age_years"),
        ("Daily VEN dose, mg/day", "daily_venlafaxine_dose_mg_day"),
        ("VEN concentration, ng/mL", "venlafaxine_concentration_ng_ml"),
        ("ODV concentration, ng/mL", "o_desmethylvenlafaxine_concentration_ng_ml"),
        ("VEN/dose, (ng/mL)/(mg/day)", "ven_dose"),
        ("ODV/VEN ratio", "odv_ven_ratio"),
    ]
    for name, col in continuous_variables:
        d = median_iqr_range(df[col])
        summary_rows.append({
            "Characteristic": name,
            "Value": f"{d['median']} [{d['q1']}-{d['q3']}] ({d['min']}-{d['max']})",
        })

    summary_rows.append({
        "Characteristic": "Age >=65 years",
        "Value": f"{int(df['age_ge_65'].sum())} ({100 * df['age_ge_65'].mean():.1f}%)",
    })
    for count in [0, 1, 2]:
        n = int((df["cyp2d6_count"] == count).sum())
        summary_rows.append({
            "Characteristic": f"CYP2D6 reduced/no-function allele count: {count}",
            "Value": f"{n} ({100 * n / len(df):.1f}%)",
        })
    pd.DataFrame(summary_rows).to_csv(outdir / "table1_summary.csv", index=False)


def create_regression_results(df, outdir):
    """Create Table 2 regression results and return selected statistics."""
    predictors = ["age_years", "cyp2d6_count"]
    results = []
    for outcome in ["ven_dose", "odv_ven_ratio"]:
        std_model = standardized_beta(df, outcome, predictors)
        y = np.log(df[outcome].astype(float))
        X = sm.add_constant(df[predictors].astype(float), has_constant="add")
        model = sm.OLS(y, X).fit()
        r2_age, _ = incremental_r2(df, outcome, predictors, "age_years")
        r2_cyp, _ = incremental_r2(df, outcome, predictors, "cyp2d6_count")
        results.append({
            "Outcome": outcome,
            "Predictor": "Age",
            "Standardized_beta": std_model.params["age_years"],
            "Multiplicative_effect": multiplicative_effect(model, "age_years", scale=10.0),
            "Effect_scale": "per 10 years",
            "P_value": model.pvalues["age_years"],
            "Incremental_R2": r2_age,
        })
        results.append({
            "Outcome": outcome,
            "Predictor": "CYP2D6 allele count",
            "Standardized_beta": std_model.params["cyp2d6_count"],
            "Multiplicative_effect": multiplicative_effect(model, "cyp2d6_count", scale=1.0),
            "Effect_scale": "per allele",
            "P_value": model.pvalues["cyp2d6_count"],
            "Incremental_R2": r2_cyp,
        })
    result_df = pd.DataFrame(results)
    result_df.to_csv(outdir / "table2_regression_results.csv", index=False)
    return result_df


def create_interaction_results(df, outdir):
    """Evaluate age-by-CYP2D6 interaction terms."""
    interaction_rows = []
    for outcome in ["ven_dose", "odv_ven_ratio"]:
        tmp = df.copy()
        tmp["age_x_cyp2d6"] = tmp["age_ge_65"] * tmp["cyp2d6_count"]
        y = np.log(tmp[outcome].astype(float))
        X = sm.add_constant(
            tmp[["age_ge_65", "cyp2d6_count", "age_x_cyp2d6"]].astype(float),
            has_constant="add",
        )
        model = sm.OLS(y, X).fit()
        interaction_rows.append({
            "Outcome": outcome,
            "Interaction_p_value": model.pvalues["age_x_cyp2d6"],
        })
    pd.DataFrame(interaction_rows).to_csv(outdir / "interaction_results.csv", index=False)


def create_sensitivity_summary(df, outdir):
    """Run sensitivity analysis excluding records flagged at both LOQ values."""
    rho, p_rho = stats.spearmanr(df["age_years"], df["ven_dose"])
    older = df.loc[df["age_ge_65"] == 1, "ven_dose"]
    younger = df.loc[df["age_ge_65"] == 0, "ven_dose"]
    mw = stats.mannwhitneyu(older, younger, alternative="two-sided")
    groups = [df.loc[df["cyp2d6_count"] == c, "odv_ven_ratio"] for c in [0, 1, 2]]
    kw = stats.kruskal(*groups)

    sens = df[df["sensitivity_excluded_both_ven_and_odv_at_loq"] == 0].copy()
    rho_s, p_s = stats.spearmanr(sens["age_years"], sens["ven_dose"])
    groups_s = [sens.loc[sens["cyp2d6_count"] == c, "odv_ven_ratio"] for c in [0, 1, 2]]
    kw_s = stats.kruskal(*groups_s)

    with open(outdir / "sensitivity_summary.txt", "w", encoding="utf-8") as f:
        f.write(f"Python version: {sys.version}\n")
        f.write(f"Platform: {platform.platform()}\n")
        f.write(f"pandas: {pd.__version__}\n")
        f.write(f"numpy: {np.__version__}\n")
        f.write(f"scipy: {scipy.__version__}\n")
        f.write(f"statsmodels: {statsmodels.__version__}\n")
        f.write(f"matplotlib: {matplotlib.__version__}\n\n")
        f.write(f"Primary N: {len(df)}\n")
        f.write(f"VEN/dose Spearman age rho={rho:.6g}, p={p_rho:.6g}\n")
        f.write(f"Mann-Whitney older vs younger VEN/dose U={mw.statistic:.6g}, p={mw.pvalue:.6g}\n")
        f.write(f"ODV/VEN Kruskal-Wallis by CYP2D6 allele count H={kw.statistic:.6g}, p={kw.pvalue:.6g}\n")
        f.write(f"Sensitivity N: {len(sens)}\n")
        f.write(f"Sensitivity VEN/dose Spearman age rho={rho_s:.6g}, p={p_s:.6g}\n")
        f.write(f"Sensitivity ODV/VEN Kruskal-Wallis H={kw_s.statistic:.6g}, p={kw_s.pvalue:.6g}\n")


def create_figure1(df, outdir):
    """Generate Figure 1 from the controlled-access analytical dataset."""
    fig, axes = plt.subplots(1, 2, figsize=(10, 4))

    axes[0].scatter(df["age_years"], df["ven_dose"], alpha=0.8)
    xs = np.linspace(df["age_years"].min(), df["age_years"].max(), 100)
    x_model = sm.add_constant(pd.DataFrame({"age_years": xs}), has_constant="add")
    simple = sm.OLS(np.log(df["ven_dose"]), sm.add_constant(df[["age_years"]], has_constant="add")).fit()
    ys = np.exp(simple.predict(x_model))
    axes[0].plot(xs, ys)
    axes[0].set_yscale("log")
    axes[0].set_xlabel("Age, years")
    axes[0].set_ylabel("VEN/dose, (ng/mL)/(mg/day)")
    axes[0].set_title("A. Parent-drug exposure")

    box_data = [df.loc[df["cyp2d6_count"] == c, "odv_ven_ratio"] for c in [0, 1, 2]]
    axes[1].boxplot(box_data, positions=[0, 1, 2], showfliers=False)
    for c, vals in zip([0, 1, 2], box_data):
        jitter = np.random.default_rng(12345 + c).normal(0, 0.04, len(vals))
        axes[1].scatter(np.full(len(vals), c) + jitter, vals, alpha=0.8)
    axes[1].set_yscale("log")
    axes[1].set_xlabel("CYP2D6 reduced/no-function allele count")
    axes[1].set_ylabel("ODV/VEN ratio")
    axes[1].set_xticks([0, 1, 2])
    axes[1].set_title("B. O-demethylation ratio")

    fig.tight_layout()
    fig.savefig(outdir / "figure1_outcome_specific_associations.png", dpi=300)


def main():
    parser = argparse.ArgumentParser(description="Reproduce manuscript analyses from the controlled-access dataset.")
    parser.add_argument(
        "--input",
        default="VEN_minimum_deidentified_analytical_dataset_controlled_access.csv",
        help="Path to the controlled-access deidentified analytical dataset.",
    )
    parser.add_argument("--outdir", default="analysis_outputs", help="Output directory.")
    args = parser.parse_args()

    outdir = Path(args.outdir)
    outdir.mkdir(parents=True, exist_ok=True)

    df = pd.read_csv(args.input)

    # Derived variables used in the manuscript.
    df["age_ge_65"] = (df["age_years"] >= 65).astype(int)
    df["ven_dose"] = df["ven_dose_ratio_ng_ml_per_mg_day"]
    df["odv_ven_ratio"] = df["odv_ven_metabolic_ratio"]
    df["cyp2d6_count"] = df["cyp2d6_reduced_no_function_allele_count"]

    create_table1(df, outdir)
    create_regression_results(df, outdir)
    create_interaction_results(df, outdir)
    create_sensitivity_summary(df, outdir)
    create_figure1(df, outdir)

    print(f"Analysis complete. Outputs written to: {outdir}")


if __name__ == "__main__":
    main()
```
