# Supplementary material for: Distinct Associations of Age and CYP2D6 Genotype With Parent Venlafaxine Exposure and O‐Demethylation in Japanese Psychiatric Patients
Source: Neuropsychopharmacol Rep. 2026 Oct 29;46(4):e70175. doi: 10.1002/npr2.70175 (PMC13719267; doi:10.1002/npr2.70175)
Supplement: Supplementary file 2 [file NPR2-46-e70175-s002.docx]

**Supplementary Table S1. Variable definitions for the controlled-access analytical dataset**

This table defines the variables used by Supplementary Code S1. It contains no patient-level data and no identifying information.

| **Variable name** | **Definition** |
| --- | --- |
| study_record_id | Sequential study-specific record number assigned for this deidentified analytical dataset; not an original patient ID. |
| age_years | Age at blood sampling or measurement in years. |
| daily_venlafaxine_dose_mg_day | Daily venlafaxine dose in mg/day at the time of blood sampling. |
| venlafaxine_concentration_ng_ml | Plasma parent venlafaxine concentration in ng/mL. Values at the lower limit of quantification are coded as 2.5 ng/mL. |
| o_desmethylvenlafaxine_concentration_ng_ml | Plasma O-desmethylvenlafaxine concentration in ng/mL. Values at the lower limit of quantification are coded as 2.5 ng/mL. |
| cyp2d6_genotype | CYP2D6 genotype available in the analytical dataset. Genotype labels are normalized for presentation, for example *1/*10. |
| cyp2d6_reduced_no_function_allele_count | Number of CYP2D6 reduced- or no-function alleles used in the manuscript analysis. CYP2D6*10 and CYP2D6*5 were counted as reduced/no-function alleles and coded as 0, 1, or 2. |
| ven_dose_ratio_ng_ml_per_mg_day | Dose-corrected parent venlafaxine exposure calculated as venlafaxine_concentration_ng_ml divided by daily_venlafaxine_dose_mg_day. |
| odv_ven_metabolic_ratio | Metabolic ratio calculated as o_desmethylvenlafaxine_concentration_ng_ml divided by venlafaxine_concentration_ng_ml. |
| sensitivity_excluded_both_ven_and_odv_at_loq | Flag identifying records excluded from the sensitivity analysis because both venlafaxine and O-desmethylvenlafaxine concentrations were at the lower limit of quantification; 1 = excluded, 0 = retained. |
